# The Causes and Prevention of Infantile Diarrhœal Diseases

**Published:** 1887-06

**Authors:** F. R. Campbell

**Affiliations:** Lecturer on Hygiene, Niagara University, Sanitary Inspector of the Board of Health, Buffalo, N. Y.


VOL. XXVI. JUNE, 1887. NO. 11

### Original Communications.

### *THE CAUSES AND PREVENTION OF INFANTILE DIARRHŒAL DISEASES.**

BY F. R. CAMPBELL, A. M., M. D.,

Lecturer on Hygiene, Niagara University,

Sanitary Inspector of the Board of Health, Buffalo, N. Y.

Jules Simon has said that we add more to the strength and prosperity of a nation by the prevention of disease and death than by the conquest of foreign territory. It is a low death-rate rather than a high birth-rate which produces a rapid increase in population. It requires France 192 years to double her population. In Prussia this is accomplished in 54 years; in England in 52 years, while Buffalo by the same method of computation will double her population from natural growth every 69 years.

Preventive medicine has made remarkable progress during the past century. Sickness has been greatly reduced, death has been robbed of a host of victims, and the average duration of life has been increased at least twenty per cent. Jenner by his immortal discovery has saved thousands from death, blindness and disfiguration. Ferran, Freire and Pasteur promise us immunity from Asiatic cholera, yellow fever and rabies. We may even hope to find a clue to the causes and cure of that dread

*Read before the Buffalo Medical and Surgical Association, June, 1887.

malady, consumption. It is to a small field in the department of preventive medicine that I wish to invite your attention this evening, promising you no new discoveries, but simply applying, as far as possible, old facts to a new locality—our own city.

My subject is "The Causes and Prevention of the Diarrhœal Disease of Infants," and it is my desire to point out the local sources of these affections and trace their relation to the mortality of infants in general.

No city in the United States has more accurate vital statistics than Buffalo. Our death reports are almost absolutely correct, while the birth returns are less defective than in most other American cities. We may therefore safely employ these statistics as the basis of some of our investigations.

During the past six years, from 1881 to 1886 inclusive, 32,769 births have been reported to the Registrar of Vital Statistics, and during the same period 10,714 deaths have occurred among those under five years of age, showing that about one-third of all children born die before reaching the sixth year of life. Moreover, 6,684, or 20 per cent. of all born, died during the first year of life; 2,041, or 12 1-7 per cent. of the children one year old, died in the second year; 946, or 6 2-10 per cent. of the children two years old, died in the third year; 550, or 4 per cent. of the children three years old, died in the fourth year, and 479, or 3½ per cent. of the children in the fifth year, died annually.

If we compare this death-rate with that of European cities, we will be forced to the conclusion that Buffalo is a remarkably healthy place for babies, if not the healthiest city of its size, in the world. We have only to compare our death-rate for children under one year of age, 20 per cent., with the rates given by Ecklund for several cities of Europe in 1872 to observe the remarkable difference:

| City. | Deaths per 100 under 1 Year. | City. | Deaths per 100 under 1 Year. |
|---|---|---|---|
| Paris, . . | 30.8 | Vienna, . . . | 42.8 |
| St. Petersburg, . | 32.5 | London, . . . | 43.5 |
| Milan, . . . | 32.5 | Dresden, . . . | 46.3 |
| Prague, . . . | 32.8 | Venice, . . | 46.5 |

| City. | Deaths per 100 under 1 Year. | City. | Deaths per 100 under 1 Year |
|---|---|---|---|
| Turin, | 32.9 | Hamburg, | 48 4 |
| Marseilles, | 35.3 | Rotterdam, | 53.6 |
| Naples, | 36.4 | Munich, | 55.5 |
| Rome, | 40.4 | Stuttgart, | 56 4 |
| Moscow, | 40.4 | Berlin, | 58.1 |

The average infant mortality for all Europe is 25.4 per cent. In the large cities of the United States with the exception of Buffalo—that is, in Boston, New York, Philadelphia, Chicago and St. Louis—the infant mortality is said to be fifty per cent, which is manifestly too great, as the birth returns in all these places are very defective. In the various departments of France where vital statistics are accurately recorded, it has been found that the mortality of infants during the first year of life varies from five per cent in the Department du Rhone to eighty-five per cent. in the Department du Loire Inferior, the average for all France being according to Monot seventeen and a-half per cent France has the lowest infant mortality on the Continent, a fact which is in part due to the low birth-rate. Great pains are taken with the few children born, and as a consequence the mortality is low; the Germans have a birth rate nearly double that of the French, but the mortality is enormously increased. A sarcastic French writer has said that "Germans like hogs are very prolific animals, and their offspring are likewise liable to die young."

French sanitarians are in the habit of considering seventeen and a-half per cent. as the normal rate of infant mortality, but Monot maintains that five per cent. is much nearer what the normal should be, as the death-rate is found to be as low as this where the best sanitary conditions are observed. In other words he claims that the French people through carelessness and ignorance destroy one-eighth of their infants during the first year of life, and we of Buffalo lose one-sixth of our infants for the same reasons.

With the hot sultry days of summer comes a slaughter of the innocents in all northern American cities far greater than that which Herod ordered, and this excessive mortality is due largely to diarrhœal affections, a class of diseases regarded as preventible by the great majority of sanitarians. The following table will show the number of deaths from diarrhœal diseases in Buffalo occurring among those under five years of age during the past six years, and the percentage of all deaths for the same time due to these causes:

| Year. | Deaths from Diarrhœal Disease under 1 year. | From 1 to 2 years. | From 2 to 3 years. | From 3 to 4 years. | From 4 to 5 years. | Total from Diarrhœal Diseases under 5 years. | Per cent. of Deaths all under 5 years from Diarrhœa. | Total Deaths under 5 years from all causes. | | |
|---|---|---|---|---|---|---|---|---|---|---|
| 1881.. | 291 | 149 | 38 | 11 | 11 | 500 | 27 | 1,851 | | |
| 1882.. | 311 | 76 | 9 | 2 | 6 | 407 | 20½ | 1,973 | | |
| 1883.. | 308 | 58 | 9 | 2 | 2 | 379 | 26 | 1,475 | | |
| 1884.. | 336 | 111 | 25 | 12 | 7 | 491 | 26 | 1,890 | | |
| 1885.. | 344 | 71 | 12 | 5 | 2 | 434 | 25 | 1,697 | | |
| 1886.. | 335 | 75 | 11 | 3 | 1 | 425 | 23½ | 1,828 | | |
| Total. | 1925 | 540 | 140 | 35 | 29 | 2,633 | 24½ | 10 714 | 45.6 of | all deaths |
| Per ct | 73 | 20½ | $\frac{9}{10}$ | 1⅓ | 1⅙ | 100 | | | | |

This table shows that there have been 2,633 deaths from diarrhœal diseases among young children in this city during the past six years, and that nearly one-fourth (24½ per cent.) of all deaths among children are due to these maladies.

To discuss the etiology of infantile diarrhœal diseases in our climate is to discuss the causes of infant mortality in general. Whatever diminishes the strength and vigor of the system tends to produce intestinal disease in summer. There are a host of causes, some of which act in a very indirect manner; but no specific cause has yet been discovered. But for the sake of a classification we may divide the factors which we are about to consider into five classes: developmental, social, meteorological, sanitary and dietetic.

### I. DEVELOPMENTAL CAUSES.

1. *Age.*—To speak of age and sex as causes of a disease is manifestly illogical, and yet these conditions have close relations to the causation of many affections. In infancy there is the greatest activity of the assimilative functions; the child attains nearly half its mature weight and stature during the first three years of life. The younger the child the greater the activity of nutrition and the less the power to resist disease. There are certain peculiarities in the digestive organs of infants which deserve a passing mention. The glycogenic ferments are not found in the saliva and pancreatic fluids until the first teeth appear, pepsin is secreted in but small quantities by the gastric glands, and the liver does not properly convert the peptones, uric acid being formed and eliminated instead of urea. Prof. Owen has shown that in the early stages of human life the functions of many organs resemble those of the lower animals. Birds and reptiles excrete uric acid instead of urea normally, owing to certain peculiarities in their hepatic organs. In the infant, the syphilitic child and the gouty man the same peculiarity is observed, and a carefully restricted diet is required. During the first two months of life the sweat glands possess but little if any functional activity. By regulating cutaneous evaporation these organs preserve the normal temperature of the body, but since their functions are so defective in infancy, sudden changes in the temperature of the surrounding atmosphere will affect the normal heat of the blood. When fluids cannot be eliminated by the skin for the reduction of temperature, copious discharges from the bowels are excited, a phenomenon which is usually attended with a loss of bodily heat.

Of the 2,633 deaths from diarrhœal diseases among children which have been reported during the past six years, 73 per cent. have been under 1 year of age, 20½ per cent. between 1 and 2 years, 4 per cent. between 2 and 3 years, 1½ per cent. between 3 and 4 years, and 1 per cent. between 4 and 5 years of age.

Furthermore, 29 per cent. of all deaths among children under 1 year of age in Buffalo are due to diarrhœal diseases; 26½ per cent. of those between 1 and 2 years of age, 10 per cent. of those between 2 and 3 years, 6½ per cent. of those between 3 and 4 years, and 6¼ of those between 4 and 5 years of age are due to the same causes, showing that not only does the mortality from these affections but also the relative proportion of all deaths attributable to them vary inversely with the age.

Dr. J. Lewis Smith observed that the mortality from diarrhœal diseases among infants from 6 to 12 months of age was double that of children under six months of age. This increased mortality during the latter half of the first year is attributed by some writers to the occurrence of dentition at this time; but the comparative immunity observed during the first six months of life is largely due to the fact that more are nursed at the mother's breast, and it is not all demonstrated that they are less liable to intestinal diseases, other circumstances being equal.

2. *Sex.*—The male child grows more rapidly than the female. The metamorphosis of tissues being greater, the liability to disease is proportionately increased and the power of resistance diminished. Nature has very kindly provided in part for this excess of male deaths by creating an excess of male births. But even with her best efforts a large number of our females must be doomed to perpetual maidenhood. During the past six years there have been 16,953 male births reported to 15,650 female births, showing that about 108 males are born to every 100 females. On the other hand, 118 males have died to every 100 females, and this great excess in the male death-rate is chiefly among children. Of the 2,633 deaths from diarrhœal diseases among children in Buffalo, 1,412 have been males and 1,221 females, showing that 115½ male children die of these affections to every 100 females, an excess of male deaths nearly double the excess of male births. Bertillon has discovered that in Paris 121 males die under one year of age to 100 females, and that this proportion is about the same in other European cities. In other words, he claims that one fifth of all boys born die in the first year of life, while but one sixth of the girls die during the same period. In Buenos Ayres where diarrhœal diseases are quite as prevalent as in Buffalo, there is about the same difference in the death-rate of the sexes from these affections as in our city.

II. SOCIAL CAUSES.

*Wealth.*—Diarrhœal affections are pre-eminently diseases of the poor. Ignorance and filth are the usual accompaniments of poverty, and as a consequence all prophylactic measures are disregarded. Bouchut observed that the infant mortality in the eleventh, twelfth and seventeenth arrondissements of Paris, occupied principally by the poor, was more than double that of the children found in the sections occupied by those in comfortable circumstances. The same fact has been observed by Dr. J. L. Smith in New York. In some of the localities occupied by the poor, when acting as a sanitary inspector, he found cases of diarrhœa among the children in almost every house. In Buffalo the death-rate from diarrhœal diseases in the Fifth Ward, where a large number of Poles are found, is 4.7 per 1,000, nearly four times greater than that of the Ninth Ward, occupied by a wealthier class.

Joseph Lefort has studied the mortality of infants in industrial centres. In many of the factories of France and England, where women are employed, the children are left in crêches during the day, while the mothers are at work. In the crêche the children were dry-nursed, and as the women were in the habit of leaving their infants there on the ninth day after confinement, the mortality was frightful, in some places reaching eighty-three per cent. But a very simple remedy was discovered, which reduced this mortality in a remarkable manner. A fund was established by the proprietor of the Mulhouse Factory, to support the mothers at home until the children were seven weeks of age. In this manner the mortality was reduced to thirty per cent., and even less in some towns. We also have our crêche and working women, but the social relations of employers and the working classes are such in this country that any control of the sort just described would be impossible.

2. *Nationality.*—As a general rule, those nationalities having the highest birth-rate have the highest infant mortality and highest death-rate from diarrhœal affections. Among the Poles and Germans diarrhœal affections are common; they are moderately frequent among English, Irish and Italians, but rare among French and Scotch. It was at one time supposed that races coming from a cold climate to America were more liable to diarrhœal affections than those who emigrate from hot countries, but statistics prove that this is not strictly true, for the Italians are not exempt from these diseases, nor are the Scotch from the north countries especially liable to them.

3. *Legitimacy.*—In France 9 per cent. of all the children born are illegitimate, while in Paris even 30 per cent. are born out of wedlock. Chauffard has made a careful study of the mortality of illegitimate children, and finds that it is about 100 per cent. greater than that of the legitimate. In Buffalo not 2 per cent. of the children are born out of wedlock, but the mortality among these is immense, as the records of lying-in asylums and "baby farms" would show. It is customary in this country to place these unfortunate babies with some woman who agrees to care for them for two or three dollars per week. For a hundred dollars many will stipulate to provide for the infant permanently, but in the great majority of cases the babies die of marasmus or enteritis before many months have elapsed. In France a bureau of wet nurses is provided by the public to care for these children. They are generally taken outside of the cities, but as each nurse cares for two or three babies, you may imagine that the wet-nursing is of a very defective character. Nevertheless, the mortality is much less than in our foundling homes where dry-nursing is the rule.

### III. METEOROLOGICAL CAUSES.

The diarrhœal diseases of children in our latitude are confined almost entirely to the hot months, ending with the first cold days of autumn. The epidemic begins in Buffalo about the 1st of July—fully two weeks later than in New York—and disappears about the middle of October. The youngest children are first affected, and if the atmospheric and other conditions are favorable those of a more advanced age are attacked; the disease in these latter cases being more of a dysenteric than choleriform character. The maximum mortality of infants from diarrhœal diseases is reached in August. The maximum from dysentery is attained a month later, in September. It is, indeed, a remarkable fact that in Buffalo dysentery makes its appearance and passes away about one month after the choleriform diarrhœa of children. In the following table is given the mortality from diarrhœal diseases by months for the six years, 1881 to 1886 inclusive, with the percentage of deaths belonging to each month:

| Months. | 1881. | 1882. | 1883. | 1884. | 1885. | 1886. | Total. | Per cent. of all. |
|---|---|---|---|---|---|---|---|---|
| January....... | 0 | 1 | 4 | 5 | 0 | 6 | 16 | 3/5 |
| February ..... | 0 | 3 | 2 | 3 | 3 | 2 | 13 | ½ |
| March......... | 0 | 4 | 7 | 4 | 4 | 1 | 20 | 4/5 |
| April ........ | 0 | 2 | 0 | 0 | 2 | 0 | 4 | 1/6 |
| May........ | 5 | 6 | 1 | 5 | 9 | 5 | 31 | 1/5 |
| June ......... | 9 | 9 | 9 | 16 | 5 | 7 | 55 | 2 |
| July. ........ | 110 | 51 | 68 | 58 | 112 | 149 | 548 | 21 |
| August......... | 121 | 171 | 169 | 193 | 142 | 123 | 919 | 35 |
| September.... | 151 | 130 | 73 | 135 | 131 | 108 | 728 | 27 |
| October....... | 93 | 26 | 35 | 64 | 26 | 15 | 259 | 9 4/5 |
| November .... | 9 | 1 | 9 | 8 | 3 | 5 | 35 | 1½ |
| December..... | 2 | 0 | 2 | 5 | 6 | 4 | 19 | 4/5 |
| Total.. ..... | 500 | 404 | 373 | 491 | 434 | 425 | 2,633 | |

The above table shows that these diseases belong almost exclusively to the third quarter of the year. They make their appearance about the first of June, but are not of a severe type until July, when the severity and frequency of the attacks rapidly increase until the maximum mortality is reached during the first few days of August.

1. *Temperature.*—Excessive heat is a prominent cause of infantile diarrhœa. Beginning in May, the curve of mortality from cholera infantum corresponds almost exactly with curve of mean temperature. Furthermore, it has been observed that the mortality curve of the summer diarrhœa of infants consists of recurring waves corresponding with the hot waves which pass over our country in the summer months. This has been demonstrated by the researches of Dr. S. Busey of Washington, in connection with the late chief signal officer, Gen. Myer. This fact has been observed in Buffalo, as shown in a paper read before the Buffalo Medical Association in 1885. But it is evidently not the high temperature alone which causes the increased mortality from diarrhœal diseases in summer. If this were the case we would find that the mortality would increase as we travel toward the equator, and that the mortality would be nearly as great in the country as in the city. New Orleans has a lower infant mortality in summer than Philadelphia, Chicago or Boston. In France the infant mortality is often higher in winter than in summer. Milne Edwards has made numerous experiments showing that cold is more injurious than heat to the health of young animals. August has been uniformly the hottest month in Buffalo, but in 1881 the greatest mortality from diarrhœal diseases occurred in September, and in 1886 in July. Sudden changes of temperature are more important in the production of diarrhœal diseases than a uniform high temperature as the investigations of Dr. Busey have shown.

This writer has made a careful study of the subject in Washington, and has arrived at the following conclusions:

(1) The month of July is the hottest and sickliest month of the year, most conducive to bowel affections and most fatal to children under five years of age.

(2) The epidemics of the bowel affections of children incident to the summer season have their beginning nearly simultaneously with the first exacerbation of heat which usually occurs in the latter half of June; the maximum daily mortalities frequently corresponding with the maximum temperatures which occur in periods of three or more days at longer or shorter intervals during the summer months.

(3) With the usual lowering of temperature and the absence of excessive heat periods which occur after August, the daily mortality declines.

(4) The detrimental influence of summer temperature is intensified by sudden and acute elevations and falls.

(5) Children under one year of age are most numerously and seriously affected by the temperature influence.

2. *Humidity.*—Paul Bert has shown that more carbon dioxide is eliminated in moist air than in dry; consequently there is greater activity of tissue changes in moist than in dry weather. Furthermore moist air is a better carrier of zymotic poisons than dry. The dry Harmattan wind which blows from the interior of Africa puts an end to all zymotic diseases, even small pox.

Now, although we have the highest mortality from diarrhœal diseases in the month when the average relative humidity is lowest, at the same time the absolute humidity and the hygrometric fluctuation is greatest. In hot weather children as well as adults are usually attacked with bowel complaints at night when the relative humidity is high. It is a common observation that cholera infantum is most fatal on hot, moist days. The moisture of the air diminishes the radiation of heat by evaporation, and at the same time increases the production of heat in the body, as is shown by the increased elimination of carbonic acid. The facilities for a careful study of the relations of humidity to disease are very imperfect. But it is quite probable that excessive moisture in the air prevents the rapid diffusion of emanations from the soil, which in hot weather in the city are of a dangerous character. It has been observed in New York and Baltimore that cholera infantum was extremely common and fatal among the children occupying basements, and it has been proposed to enact laws prohibiting landlords from renting basements for dwelling purposes.

3. *Rainfall.*—Rain is a great purifier of the air and soil. The great epidemics of the middle ages almost uniformly followed droughts. The highest death-rate in England during the past twenty-five years occurred in 1864, the year in which the rainfall was lowest. The highest mortality from diarrhœal diseases in Buffalo occurred in 1881, the year in which there was a drought. During six weeks in August and September less than a quarter of an inch of rain fell, the atmosphere became hazy, vegetation was withered and dry, the soil was parched to a depth of five feet, the wells were dried, and in the thicklysettled unsewered districts a disagreeable odor was everywhere observed. The infant mortality from diarrhœal diseases increased with the drought; in June there were nine deaths, July 110, August 121, September 151, October 93. Moreover, as the drought increased, the bowel affections assumed more of a dysenteric character. A fact particularly noticeable was that it was the children of more than one year of age who were chiefly affected by the drought. The mortality from diarrhœal diseases among those over one year of age was fifty per cent. higher than in any other year, whereas the mortality of those under one year of age was not increased. We may conclude then:

(1) That a drought in hot weather will increase the mortality from diarrhœal diseases.

(2) That the increased mortality is found chiefly among those over one year of age.

(3) That the diarrhœal disease occurring during a drought is liable to be of a dysenteric character.

(4) That the increased mortality is most marked in localities where sewerage is defective and the water supply derived from wells, as is shown by the fact that dysentery was most prevalent in the unimproved sections of the city.

4. *Velocity of wind.*—The movement of the air currents is more closely related to the prevalence of diarrhœal affections than is usually supposed. Running water will purify itself; in like manner the air is purified by diffusing and oxidising the injurious substances held in suspension. In Buffalo the maximum mortality from diarrhœal diseases occurs in the month when the movement of the winds is least. The National Board of Health discovered that this was true of twenty-seven cities investigated by them in regard to the prevalence of cholera infantum, San Francisco being the only exception. An extremely varying velocity of wind in hot weather will increase diarrhœal affections. In San Francisco the velocity is uniformly high, and the death-rate from infantile diarrhœal diseases is but 5 in 10,000; in New Orleans the velocity is uniformly low, and the death-rate is but 7 in 10,000. But in Buffalo, Boston,

Brooklyn and Chicago the velocity of the wind is extremely variable, and we find a mortality from diarrhœal affections amounting to 23, 24, 25 and 34 in 10,000 respectively.

The National Board of Health has reached the following conclusions regarding the relation of the velocity of the wind to diarrhœal affections:

(1) That the greatest mortality from diarrhœal diseases occurs in the month when the movement of the wind is least.

(2) The nearer the monthly movement of the wind approaches uniformity, the less the mortality from summer diarrhœa.

5. *Ozone.*—In Michigan the health authorities have studied the relation of atmospheric ozone to various diseases, and some very interesting phenomena have been observed, viz.: that maximum of acute respiratory diseases occurs in months when there is the maximum of atmospheric ozone, and that the maximum of acute intestinal diseases occurs when there is least atmospheric ozone. It has been recently demonstrated that the amount of atmospheric ozone is an index of the purity of the air. It is abundant at sea and in the mountains; it is wanting in cities during hot months and wherever there is abundant decomposing matter. When we discover, therefore, that there is most diarrhœal disease when there is least atmospheric ozone, the real meaning is that the air is most impure.

To sum up the relation of meteorological conditions to the prevalence of infantile diarrhœa we will state—

(1) That when there is more than the average of diarrhœal diseases the average daily range of temperature, the average temperature and the absolute humidity of the atmosphere are *greater* than the average for the year, and vice versa.

(2) That when there is more than the average of diarrhœal diseases, the relative humidity of the air, the rainfall, the average velocity of the wind, and the amount of ozone are less than the average for the year, and vice versa.

How does hot weather induce diarrhœa in children? The answer to this question hinges largely on the fact that the perspiratory functions of the infant are very defective or entirely wanting. Tardieu, Becher and Brown-Sequard have made experiments showing that the temperature of the adult body rises one degree for every twenty degrees sudden rise in the temperature of the surrounding atmosphere. In children the effect of atmospheric heat must be far greater. As perspiration is defective, nature attempts to maintain the normal heat by copious watery discharges from the bowels, a phenomenon attended by reduction of the temperature of the blood, and sometimes called sudoral diarrhœa. The digestive functions, moreover, are interfered with by the fever produced by heat, as it is a well-known fact that all fever patients are dyspeptic. But the principal action of the atmospheric heat is in the promotion of the decomposition of organic matter—filth—thus poisoning the air which the children are obliged to breathe.

(*To be continued.*)

